# Promoting an active form of learning out-of-class via answering online “study questions” leads to higher than expected exam scores in General Biology

**DOI:** 10.7717/peerj.1322

**Published:** 2015-10-08

**Authors:** Susan I. Gibson

**Affiliations:** Department of Plant Biology, University of Minnesota—Twin Cities, Saint Paul, MN, United States of America

**Keywords:** Active learning, Higher education, STEM disciplines, General Biology, Out-of-class active learning, Quizzing, Study questions, Answering questions

## Abstract

A rising need for workers in science, technology, engineering and mathematics (STEM) fields has fueled interest in improving teaching within STEM disciplines. Numerous studies have demonstrated the benefits of active learning approaches on student learning outcomes. However, many of these studies have been conducted in experimental, rather than real-life class, settings. In addition, most of these studies have focused on in-class active learning exercises. This study tested the effects of answering questions outside of class on exam performance for General Biology students at the University of Minnesota. An online database of 1,020 multiple-choice questions covering material from the first half of the course was generated. Students in seven course sections (with an average of ∼265 students per section) were given unlimited access to the online study questions. These students made extensive use of the online questions, with students answering an average of 1,323 questions covering material from the half of the semester for which the questions were available. After students answered a set of questions, they were shown the correct answers for those questions. More specific feedback describing how to arrive at the correct answer was provided for the 73% of the questions for which the correct answers were not deemed to be self-explanatory. The extent to which access to the online study questions improved student learning outcomes was assessed by comparing the performance on exam questions of students in the seven course sections with access to the online study questions with the performance of students in course sections without access to the online study questions. Student performance was analyzed for a total of 89 different exams questions that were not included in the study questions, but that covered the same material covered by the study questions. Each of these 89 questions was used on one to five exams given to students in course sections that had access to the online study questions and on three to 77 exams given to students in sections that lacked such access. Data from over 1,800 students in sections with access to the online study questions show that those students scored a statistically significant average of 6.6% points higher on the exam questions analyzed than students in sections without access to the study questions. This difference was greater than the average amount necessary to raise students’ exam grades by one grade (e.g., from a “B-” to a “B”). In addition, there was a higher correlation between number of questions answered and success on exam questions on material related to the study questions than between number of questions answered and success on exam questions on material unrelated to the study questions. The online study question system required substantial effort to set up, but required minimal effort to maintain and was effective in significantly raising average exam scores for even very large course sections.

## Introduction

Due to a rising need for highly trained workers in science, technology, engineering and mathematics (STEM) fields (PCAST STEM Undergraduate Working Group, 2012), and for an electorate better able to make informed decisions regarding complex scientific and technical issues, there is an increasing need to educate students more effectively in STEM disciplines. Answering questions has been shown to be a particularly effective learning mechanism that promotes long-term learning in laboratory studies (reviewed in Brame & Biel, 2015). Studies have also shown that answering questions improves not only rote learning, but also increases students’ ability to draw conclusions by synthesizing multiple facts (Karpicke & Blunt, 2011; Smith & Karpicke, 2014). Different question formats have been found to be effective in improving student-learning outcomes. For example, one study found little to no advantage of answering short-answer or mixed-format questions over answering multiple-choice questions in three out of four experiments (Smith & Karpicke, 2014). However, most of these studies were done in a laboratory environment (Brame & Biel, 2015). Only a limited number of studies (for example, Leeming, 2002; Larsen, Butler & Roediger, 2009) have demonstrated the efficacy of repeated testing on student performance in real-life educational settings. A small number of studies have also examined the utility of out-of-class testing as a learning strategy. In one such study, students took one online quiz per week (multiple attempts were encouraged for each quiz) for three weeks and then took an exam. Students were found to perform better on exam questions related to material covered by the quiz questions than on exam questions on material not covered by the quiz questions (McDaniel, Wildman & Anderson, 2012). In another study, students took ten online quizzes, each with ten questions, over the course of the semester. Students who took all of the quizzes were found to perform significantly better on an exam than average students from the same class (Orr & Foster, 2013).

Although intriguing, these studies leave open the question of the extent to which providing students with access to study questions can improve overall student performance in a real classroom. To address this question, students in seven sections of BIOL 1009 General Biology at the University of Minnesota Twin Cities were provided with unlimited access to an online study question database. This online database contains 1,020 multiple-choice questions on material taught during the first half (14 lectures) of the course. After students answered a set of questions, they were shown the correct answers for those questions. More specific feedback describing how to arrive at the correct answers was provided for the 73% of the questions for which the correct answers were not deemed to be self-explanatory. The effects of access to the online study questions were assessed by comparing the performance of students with access to the online study questions to the performance of students without access to the online study questions on in-class exam questions. Use of the online study questions was found to significantly improve student performance on exams.

## Materials & Methods

### BIOL 1009 General Biology course

BIOL 1009 General Biology is a one-semester (14–15 week) course. It is taught in multiple sections each term on the University of Minnesota Twin Cities campus. This course is intended for students who are not within the College of Biological Sciences, but who do have a specific need to learn biology (e.g., students intending careers in the health sciences). Course sections meet twice per week for 75 min each time. Course sections meet an additional time each week for a 2-h laboratory session. The course as a whole (lecture plus lab) is a four-credit course. Most course instructors, including the author, teach the course using primarily a traditional lecture format, but include some in-class active learning exercises as well. These in-class active learning exercises may include answering a few questions, think-pair-share or similar activities. All instructors for BIOL 1009 use the same textbook (Campbell Biology). The author taught the first half (lectures one through 14) of BIOL 1009 section 30 in 2012 and sections 1 and 30 in 2013–2015. The sections taught by the author had approximately 220 to 320 students per section.

### Online question bank

An online question bank was developed by the author for the first half of BIOL 1009 General Biology in 2012. This online question bank was developed in Moodle, an online learning platform (https://moodle.org/). The question bank contains a total of 1,020 questions covering material taught by the author during the first 14 lectures (= half) of BIOL 1009 General Biology. There are an average of 73 questions per lecture, with a range of 47–98. All of the questions in the question bank are multiple-choice questions. Each question has a single “best” answer for which full credit is assigned and three distractors for which no credit is assigned. Most of the questions fit into one of the three lower levels (remembering, understanding and applying) of a revised version (Anderson et al., 2001) of Bloom’s anatomy (Bloom & Krathwohl, 1956). A smaller number of the questions test analyzing or evaluating. Approximately half of the questions were written by the author. Most of the remaining questions were adapted by the author from questions on the MasteringBiology website (http://www.pearsonmylabandmastering.com/northamerica/masteringbiology/) that cover material from Campbell Biology Ninth Edition (Reece et al., 2011). The correct answers are indicated for all of the questions. More detailed feedback was provided by the author for the 73% of the questions for which the correct answers were not deemed to be self-explanatory. This feedback includes descriptions of how to arrive at the correct answer and/or explanations of why particular distractors are incorrect.

### Online study questions

The quiz generating function of Moodle was used to produce sets of online study questions for each of the first 14 course lectures. The sets of study questions were set up so that each time a student chose to answer a set of study questions for a particular lecture, ten questions were randomly chosen from amongst the questions in the online question bank that pertained to that lecture. Sets of study questions were also arranged to cover material from lectures 1–3, 1–5, 1–8, 1–14, 9–10, 9–12 and 9–14. The number of questions per set for sets covering more than one lecture ranged from 10 to 27. After a student finished answering a set of study questions, the student was shown a page indicating which questions the student had answered correctly or incorrectly and showing the correct answer for each question. More detailed feedback was provided for the 73% of the questions for which the correct answers were not self explanatory. The students had unlimited online access to the sets of study questions throughout the semester. The students received no credit towards their course grade for answering sets of study questions, but were encouraged to make frequent use of them.

To determine the number of questions answered by each student, excel files indicating the number of times each student in a particular course section answered questions from a particular set of study or quiz questions were downloaded from Moodle. Columns were added to each file indicating the number of questions in that set of study or quiz questions. The excel files were then combined into a single excel file and sorted by student name. The numbers of questions answered were then added together and recorded for each student.

### Online quizzes

The same question bank used for the online study questions was also used to generate online quizzes on Moodle. There were seven quizzes covering material from the first half of the course in 2012 and 2013 and ten quizzes in 2014 and 2015. To receive credit for a particular quiz, students had to complete the quiz before a deadline. Deadlines were typically one to two weeks after the last class session covering material included on that quiz. Quizzes typically had 24 questions. These questions were randomly chosen from amongst all the questions in the online question bank on material covered by that quiz. Once started, a quiz needed to be completed within 1 h. Immediately after submitting each quiz, students were shown a page indicating the correct answers for each question on their quiz and providing more detailed feedback for 73% of the questions. Students were allowed to use any resources (notes, textbooks, the web) in answering quiz questions. Students were allowed to attempt each quiz a maximum of three times, with only their highest score for each quiz being counted. In addition, the lowest two quiz scores were dropped for each student. These quizzes, as a whole, accounted for 10% of students’ course grades for the first half of the semester, or 5% for the course as a whole.

### Biology Program exam question database

The Biology Program at the University of Minnesota Twin Cities established a database of exam questions for BIOL 1009 General Biology in fall 1978. Each term new questions are added to this database by the different faculty members teaching the course. This database currently contains 2,284 active questions covering material from the entire course. Records are maintained for each question. These records indicate each course section for which that question was used on an exam and the percentage of students that answered the question correctly each time it was used on an exam. Using this information, an “expected” exam score can be calculated for each group of questions chosen from the exam question database. These “expected” exam scores are calculated by averaging the average facility indexes for the chosen questions. For this work, “expected” scores were calculated using only results from questions that had been used at least three times in exams for course sections for which online study questions were not available, and that were not honors sections. There were a total of 89 such questions. On average, the questions from the exam question database analyzed as part of this work were used in 24.8 exams for course sections for which online study questions were not available and that were not honors sections (Table S1). In total, 34 different course instructors (including the author) used one or more of the questions analyzed on an exam.

### Exams

There were three course exams. All exams took place in class and lasted for a maximum of 75 min (exams 1 and 2) or 120 min (final exam). Students were not allowed to use any resources (notes, textbooks, etc.) during exams. All exams consisted of multiple-choice questions with one “best” answer and three distractors. There were 40 questions each for exams 1 and 2 and 50 questions for the final exam. Exams 1 and 2 each accounted for 16.67% of students’ final course grades and the final exam accounted for 20.83% of final course grades. Exam 1 covered material from lectures one through eight. Questions for exam 1 were drawn from two sources. For each exam 1, 20–21 questions were chosen by the author from the online study questions. The remaining 19–20 questions (Table S1) were chosen by the author from the exam question database maintained by the Biology Program at the University of Minnesota. Both groups of questions covered material from lectures one through eight. None of the questions chosen from the exam question database were written by the author. In contrast, approximately half of the questions chosen from the study questions were written by the author. Exam 2 covered material from lectures nine through 17 (2012 and 2013), nine through 18 (2014) or nine through 19 (2015). Study and quiz questions were available only for lectures one through 14 until 2015, when study questions became available for the entire course. Questions on exam 2 can be divided into three groups. Each year the first group of 13–15 exam 2 questions was chosen by the author from amongst the study questions covering material from lectures nine through 14. The second group of 11–13 questions (Table S1) was chosen by the author from amongst the questions in the Biology Program exam question database that cover material from lectures nine through 14. The third group of 13–16 questions was chosen by the faculty member who taught the second half of the course and covered material from lectures 15 through 17 (2012 and 2013), 15 through 18 (2014) or 15 through 19 (2015).

### Ethics statement

The University of Minnesota Twin Cities IRB: Human Subjects Committee determined that the referenced study (study number 1508E77489) is exempt from review under federal guidelines 45 CFR Part 46.101(b) category #4 EXISTING DATA; RECORDS REVIEW; PATHOLOGICAL SPECIMENS. The author received this exempt study notification from the IRB via email.

### Statistical analyses

“Actual” versus “expected” scores for questions chosen from the Biology Program exam question database were analyzed using a two-tailed, paired Student’s *t*-test. Correlations between numbers of questions answered versus performance on different groups of exam 2 questions were calculated as the Pearson product moment correlation coefficient, *r*. Calculations of the *p*-values for different *r* values were performed using an online calculator (http://www.socscistatistics.com/pvalues/pearsondistribution.aspx).

## Results and Discussion

### Students made extensive use of online questions

A database of 1,020 questions covering material from the first half (first 14 lectures) of BIOL 1009 General Biology was developed in 2012. These questions were made available online to students in seven course sections from 2012 to 2015. Students answered questions from this database both as sets of optional online “study questions” and as mandatory online quizzes. Immediately after answering a set of study questions or a quiz, students were shown the correct answers for each question. Questions they had answered incorrectly were also indicated. More detailed feedback explaining how to arrive at the correct answer or why a particular distractor is incorrect was provided for 73% of the questions. The answers for the remaining 27% of the questions were self-explanatory. This form of delayed feedback (i.e., feedback received after a student has completed answering all of the questions) has been shown to be particularly effective in promoting learning (Butler & Roediger, 2008). A concern in developing a study question database that students can access outside of class is whether students will make sufficient use of the database to justify the effort of generating it. Therefore, incentives were provided to encourage students to make use of the study questions. First, students were informed that all quiz questions would be taken directly from the same online question bank as the study questions. Together these quizzes accounted for 5% of final course grades. Secondly, students were told that 50% of the exam questions covering material from the first half of the course would be chosen from the study questions. Together these exams accounted for 54% of final course grades. Students were also informed at the beginning of the semester that answering questions has been shown to be a particularly effective means of learning new material. To help emphasize that the online questions were intended primarily to help students learn the material, rather than as a means of knowledge assessment, beginning in 2014 the sets of online questions were labeled as “study questions” rather than as “practice quizzes.”

From 2012 to 2015, students answered an average of approximately 1,323 questions (an average of 94.5 questions per lecture) for the 14 lectures for which online questions were available (Table 1). As a result, during the first part of the semester students spent an average of approximately 2 h per week answering questions outside of class. This amount of time was similar to the amount of time students spent in class during the same time period. Thus, answering online questions promoted an amount of time spent on a relatively active form of learning that was roughly equivalent to devoting almost 100% of every class session to active learning exercises.

**Table 1:** Numbers of questions answered in preparation for exams 1 and 2. The average numbers of questions (study and quiz questions combined) answered per student per lecture in preparation for exams 1 and 2 are indicated. There were an average of approximately 265 students per course section.

Year, section	Exam 1	Exam 2	Mean of exams 1 + 2
2012, 30	88.7	98.3	92.8
2013, 1	89.1	94.0	91.2
2013, 30	88.6	91.1	89.7
2014, 1 and 30	95.3	105.7	99.8
2015, 1 and 30	104.9	83.6	95.7
Mean, all 7 sections	95.0	93.8	94.5

**Figure 1:**
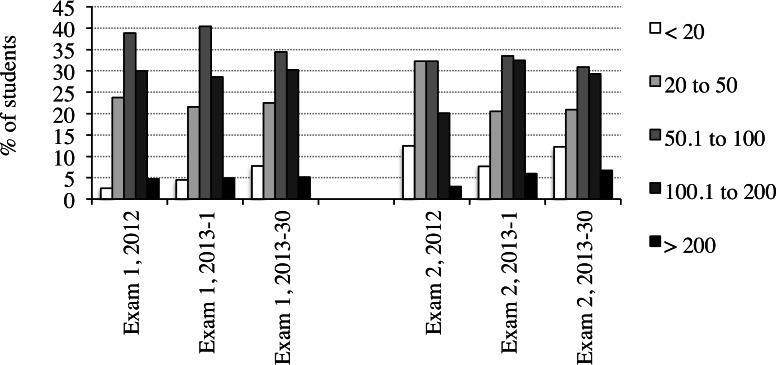
Percentages of students answering different numbers of questions per lecture. The percentages of students that answered different numbers of study and quiz questions per lecture in preparation for exams 1 and 2 in 2012 and 2013 (sections 1 and 30) are indicated. There were an average of approximately 290 students per course section.

The majority of the questions answered were optional study questions for which no credit was available, rather than quiz questions for which credit towards final course grades was available. In 2012, for example, 76% of the questions answered by students were study questions (Table 2, Table S2). The average number of questions answered per student per lecture did not vary substantially from year to year. The number of questions answered ranged from a low of 89.7 to a high of 99.8. There was a large degree of variation in the numbers of questions answered by different students. However, the vast majority of students made extensive use of the online questions. For example, in 2012 and 2013 fewer than 8% of students answered less than an average of 20 questions per lecture (Fig. 1, Table S3). In contrast, 35% of students answered an average of 50.1–100 questions per lecture and 33.6% of students answered over 100 questions per lecture. The average numbers of questions answered per student per lecture in preparation for exams 1 and 2 were very similar (Table 1). This result indicates that students’ motivation for answering large numbers of questions was maintained over the half of the semester for which questions were available. Together these results indicate that students can be motivated to make extensive use of online study questions outside of class.

**Table 2:** Numbers of study questions versus quiz questions answered in preparation for exams 1 and 2 in 2012. The average numbers of study questions and quiz questions answered per student per lecture in 2012 in preparation for exams 1 and 2 are indicated. *N* = 273.

	Exam 1, 2012	Exam 2, 2012
Study questions	64.9	77.9
Quiz questions	23.8	20.4
Total questions	88.7	98.3

**Table 3:** Correlations between numbers of online questions answered and scores on different groups of exam 2 questions. In 2012 through 2014, questions for exam 2 were drawn from three sources. For each exam 2 during those years, 13–15 questions were chosen by the author from the online study questions (=“Study questions”). Each year, an additional 11–13 questions that covered the same course material covered by the online study questions were chosen by the author from questions in the exam question database maintained by the Biology Program at the University of Minnesota (=“Questions on same material”). The final group of 13–16 questions was chosen each year by another faculty member, and covered material taught by that faculty member that was not covered by the online study questions (=“Questions on other material”). “All questions” refers to all of the 40 questions included on each exam 2. For 2013 and 2014, data from both sections taught that year were combined. The numbers of students on which each analysis was based are indicated. *r*, the Pearson product moment correlation coefficient between the numbers of online questions answered by each student in preparation for exam 2 and the student’s scores on each of the three groups of exam 2 questions. *p*, the *p*-value for the indicated *r* values.

	Study questions		Questions on same material		Questions on other material		All questions		
Year	*r*	*p*	*r*	*p*	*r*	*p*	*r*	*p*	# of students
2012	0.36	<0.00001	0.22	0.00015	0.11	0.067	0.28	<0.00001	282
2013	0.46	<0.00001	0.25	<0.00001	0.24	<0.00001	0.39	<0.00001	585
2014	0.41	<0.00001	0.31	<0.00001	0.28	<0.00001	0.39	<0.00001	477
2012–2014	0.41	<0.00001	0.27	<0.00001	0.21	<0.00001	0.36	<0.00001	1,344

### Exam scores were significantly higher than expected in course sections with access to online study questions

The Biology Program at the University of Minnesota maintains a database of questions that have been used on BIOL 1009 General Biology exams. Records are kept for each question in the exam question database. These records indicate the exams on which the question was used and the fraction of students who answered the question correctly (the facility index) on each exam. Using this information, it is possible to calculate an expected exam score for questions chosen from this exam question database. For the seven sections of BIOL 1009 that are the focus of this study, 19–20 questions from the Biology Program database were chosen for exam 1 each year and 10–13 questions were chosen for exam 2 each year. The author used the same exams for both sections of the course taught in the same year. In addition, some questions were used on exams in more than one year. To help ensure the accuracy of the results, only questions that had been used by a minimum of three course sections for which online study questions were not available (and that were not honors sections of the course) were analyzed. Consequently, a total of 89 questions (50 exam 1 and 39 exam 2 questions) chosen from the Biology Program exam question database were analyzed. These questions were used on an average of 24.8 exams, with a range of 3–77 exams, for courses for which study questions were not available (Table S1).

**Figure 2:**
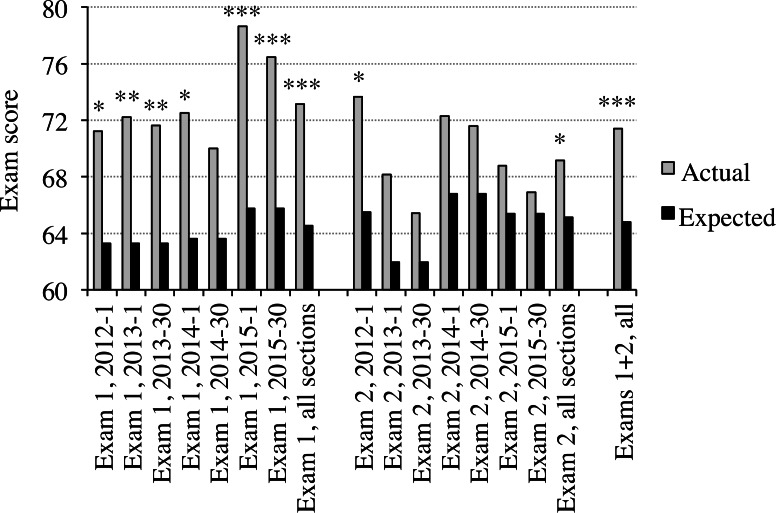
Actual versus expected partial exam scores. Some of the questions on each of the above exams were chosen from an exam question database maintained by the Biology Program at the University of Minnesota. Records are kept for each of these exam questions, indicating the percentages of students that answered each question correctly on each exam on which each question was used. Using this information, the average expected scores (based on the performance of students in course sections for which online study questions were not available) for the questions chosen from the Biology Program exam question bank are indicated by the black columns. The average actual scores (of students in course sections for which online study questions were available) for the same groups of questions are indicated by the light grey columns. The average actual and expected scores for all questions used for the first exams for all seven course sections combined are also indicated, as well as the average scores for exam 2 from all seven sections and for exams 1 and 2 combined for all seven sections. The questions analyzed were used a minimum of three times and an average of 24.8 times on exams administered to students in course sections for which online study questions were not available. The numbers of questions analyzed for each exam were 18–20 for exam 1 from each year and 10–12 for exam 2 from each year. Note that the same questions were used for exam 1 in 2012 and 2013. Also, both course sections taught in a particular year used the same exams. As a result, a total of 50 exam 1 and 39 exam 2 questions were analyzed for all seven course sections combined (Table S1). There were an average of approximately 265 students per course section. A paired Student’s *t*-test was used to compare the expected and actual scores for the questions from the Biology Program exam question database for each of the indicated exams or groups of exams. *P* values are indicated as follows: ^∗^ = < 0.05, ^∗∗^ = < 0.01 and ^∗∗∗^ = < 0.002.

The actual scores achieved by students in the seven course sections for which online study questions were available were consistently higher than expected (Fig. 2, Table S1). The combined average actual score on exam 1 for the seven course sections for which online study questions were available was 73.2%. The average expected score for these same exams, based on the percentages of students who answered each question correctly on exams administered to BIOL 1009 course sections for which online study questions were not available, was 64.5%. Therefore, students from course sections where online study questions were available scored 8.7% points higher, on average, on exam 1 than students from course sections for which online study questions were not available. The differences between the actual and expected scores on exam 2 were typically less than for exam 1. However, these differences were still statistically significant when data for exam 2 from all seven course sections for which online study questions were available are combined (Fig. 2). The average increase in exam scores for students in sections for which online study questions were available relative to students in sections for which online study questions were not available was 6.6% points for exams 1 and 2 combined. The average gap between course grades in BIOL 1009 was 5% points. Thus, this increase in exam scores was greater than the average increase needed to raise a student’s exam scores one grade (e.g., from a B- to a B).

### Answering study questions positively correlates with exam scores

The above analyses indicate that course sections for which online study questions were available had higher exam scores than sections for which online questions were not available. The results of additional analyses indicate that number of questions answered positively correlates with exam performance. For 2012–2014, a comparison of the number of questions answered in preparation for exam 2 versus score on exam 2 showed a correlation coefficient (*r*) of 0.36 (Table 3, Table S4). Of course, this high correlation coefficient could simply indicate that studying in general improves exam scores. To examine the effects of answering questions on exam scores more closely, students’ scores on exam 2 were calculated separately for each group of questions on exam 2. The first group of questions on exam 2 for 2012–2014 consisted of 13–15 questions that were chosen by the author from the online study questions. The second group of exam 2 questions during 2012–2014 consisted of 11–13 questions that were chosen by the author from questions in the exam question database maintained by the Biology Program at the University of Minnesota and that covered the same material covered by the online study questions. Thus, this second group of questions covered the same material as the first group of questions. However, unlike the first group of questions, the second group of questions were not written by the author and had not been seen previously by the students in the study or quiz questions. The third group of questions on exam 2 from 2012 to 2014 consisted of 13–16 questions that were chosen by another faculty member and that covered different material from that covered by the study and quiz questions. Not surprisingly, the highest correlation between number of online questions answered and success on a group of exam 2 questions was for the group of exam 2 questions drawn directly from the study questions (Table 3). Interestingly, there was a somewhat higher correlation between number of questions answered and success on the group 2 questions (those that covered the same material as the study questions but that were not study questions) than between number of questions answered and success on group 3 questions (those that covered different material than the study questions). This result suggests that the correlation between number of questions answered and success on the exams is not fully explained by students who tend to study more doing better on exams.

## Conclusions

Students made extensive use of the online study questions. Students answered an average of approximately 1,323 questions, or 94.5 questions per lecture, over the half of the semester for which study questions were available. As a result, students spent an amount of time on a relatively active form of learning outside of class that was approximately equal to the total amount of time spent in class during this period. The availability of the online study questions significantly raised students’ test scores. Students in course sections for which online study questions were available scored an average of 6.6% points higher on exam questions than students in sections for which online study questions were not available. This increase in exam scores was greater than the average amount needed to raise a student’s exam score by one grade (e.g., from a “B−” to a “B” or a “B” to a “B+”). This study therefore indicates the desirability of providing students with opportunities for more active forms of learning outside of class. The online study question system analyzed in this work provides an effective means of achieving this goal. It requires substantial effort to set up in the first year, but requires minimal effort to maintain in subsequent years. In addition, it is effective in significantly raising average exam scores for even very large course sections.

## Supplemental Information

10.7717/peerj.1322/supp-1 Table S1 Actual versus expected scores on exam questions from the University of Minnesota Biology Program exam question database This table summarizes information on the exam questions from the University of Minnesota Biology Program exam question database for BIOL 1009 that were analyzed as part of this work. “Mean actual score” refers to the percentage of students who correctly answered the indicated question on the indicated exam for the indicated year and course section. “Mean expected score” refers to the percentage of students who correctly answered the indicated question on all exams in which the question was used, excluding exams from one of the seven course sections being analyzed as part of this work (i.e., course sections for which online study questions were available) and from honor’s sections. ”# of other exams on which question was used” refers to the number of times the indicated question has been used on a BIOL 1009 exam at the University of Minnesota, excluding exams from one of the seven course sections being analyzed as part of this work and exams from honor’s sections of BIOL 1009.

10.7717/peerj.1322/supp-2 Table S2 Numbers of study and quiz questions answered and exam scores in 2012 The numbers of study questions, quiz questions and total questions (study plus quiz questions) answered by each student in preparation for exams 1 and 2 during 2012 are shown. The overall exam 1 and 2 scores for each student are also indicated. In addition, the scores on different groups of exam 2 questions are shown. “Exam 2 study questions” refers to questions on exam 2 that were taken directly from the online study questions. “Questions on same material” refers to questions covering the same material covered by the online study questions; these questions were chosen by the author from the exam question database maintained by the Biology Program at the University of Minnesota (thus these questions were not written by the author). “Questions on other material” refer to questions on material not covered by the study questions; these questions were chosen by a different faculty member.

10.7717/peerj.1322/supp-3 Table S3 Total numbers of questions answered in preparation for exams 1 and 2 in 2012–2013 The total numbers of questions (study questions plus quiz questions) answered by each student in preparation for exams 1 and 2 in 2012 and 2013 are indicated. The average number of questions answered per lecture for each student in preparation for exams 1 and 2 are also shown. The questions in preparation for exam 1 covered material from eight lectures and the questions in preparation for exam 2 covered material from six lectures.

10.7717/peerj.1322/supp-4 Table S4 Number of questions answered versus scores on different groups of exam 2 questions In 2012 through 2014, questions for exam 2 were drawn from three sources. 13–15 questions were chosen by the author from the online study questions, 11–13 questions that covered the same course material covered by the online study questions were chosen by the author from questions in the exam question database maintained by the Biology Program at the University of Minnesota (=“Questions on same material”) and 13–16 questions were chosen by another faculty member and covered material taught by that faculty member that was not covered by the online study questions (=“Questions on other material”). The number of questions answered by each student in preparation for exam 2 and the scores obtained by that student on the three groups of exam 2 questions and exam 2 overall are indicated.
